# Vaccination and Transmission Risk during the Outbreak of B.1.1.529 (Omicron)

**DOI:** 10.3390/vaccines10071003

**Published:** 2022-06-23

**Authors:** Barbara Grüne, Jakob Grüne, Annelene Kossow, Christine Joisten

**Affiliations:** 1 Public Health Department Cologne, Infektions- und Umwelthygiene, 50667 Köln, Germany; barbara.gruene@stadt-koeln.de (B.G.); jakob.gruene@stadt-koeln.de (J.G.); annelene.kossow@stadt-koeln.de (A.K.); 2 Institute of Hygiene, University Hospital Muenster, Albert-Schweitzer-Campus 1, 48149 Münster, Germany; 3 Department for Physical Activity in Public Health, Institute of Movement and Neurosciences, Am Sportpark Müngersdorf 6, German Sport University Cologne, 50933 Cologne, Germany

**Keywords:** booster vaccination, unvaccinated infected person, transmission, Omicron variant

## Abstract

Since its first description in November 2021, the SARS-CoV-2 variant of concern Omicron (B.1.1.529) has emerged as the dominant strain in the COVID-19 pandemic. To date, it remains unclear if boosted vaccination protects against transmission. Using data from the largest German Public Health Department, Cologne, we analyzed breakthrough infections in booster-vaccinated infected persons (IP; booster-vaccinated group (BVG); *n* = 202) and fully vaccinated, not boosted SARS-COV2-positive patients (>3 month after receiving the second dose; unboosted, fully vaccinated group (FVG); *n* = 202) to close contacts compared to an age- and sex-matched unvaccinated control group (UCG; *n* = 202). On average, IPs had 0.42 ± 0.52 infected contacts in relation to the total number of contacts in the BVG vs. 0.57 ± 0.44 in the FVG vs. 0.56 ± 0.43 in the UVG (*p* = 0.054). In the median test, pairwise comparison revealed a significant difference between the BVG and both other groups; no difference was found between the fully vaccinated and the unvaccinated control group. Now, these findings must be verified in larger samples, considering the role of Omicron subvariants and the vaccination status of the contact person. However, the importance of the booster vaccination in breaking possible chains of infection in the immune escape variant Omicron is obvious.

## 1. Introduction

In November 2021, the SARS-CoV-2 variant of concern Omicron (B.1.1.529) was first described in South Africa and Botswana [1]. Meanwhile several Omicron lineages have been found: BA.1/B.1.1.529.1, BA.1.1/B.1.1.529.1.1, BA.2/B.1.1.529.2 and BA.3/B.1.1.529.3. Omicron and its subvariant are characterized by a milder course, but higher infectivity due to the high number of more than 30 amino acid mutations within the spike protein, 15 of which occur in the receptor-binding domain (RBD; [2]). These mutations seem to be associated a higher positive electrostatic surface potential increasing the interaction between RBD and electronegative human angiotensin-converting enzyme 2 [2]. Consequently, Omicron rapidly spread in regions with high levels of population immunity and has now emerged as the dominant strain in the COVID-19 pandemic.

Besides infectivity, the immune escape capability of Omicron has also been concerning since it may elevate reinfection rates and be less sensitive to neutralizing antibodies [3]. Planas et al. [4] showed that Omicron significantly impacted most of the neutralizing potency of therapeutic monoclonal antibodies, and therefore reduced the neutralization activity of BNT162b2 and Vaxzevria convalescent sera, 5 months after full vaccination. This might partially be explained by the natural decline in humoral response over time [5,6,7]. Therefore, a booster dose of BNT162b2 triggered strong immunity against Omicron. Nevertheless, evidence of preserved CD8+ T-cell immunity against Omicron has been observed [8]. Hansen et al. [9] showed in a Danish cohort study that BNT162b2 or mRNA-1273 primary vaccine protection against Omicron decreases quickly over time. On the other hand, the booster vaccination offered a significant increase in protection. 

Puhach et al. [10] investigated the viral load in SARS-CoV-2-infected individuals during the first five days of symptoms using an in vitro culturability assay in unvaccinated or vaccinated individuals infected with the wild type (Wuhan-H-1), Delta or Omicron. Full vaccination (defined as >2 weeks after receiving the second dose during the primary vaccination series) reduced infectious viral load in Delta breakthrough cases compared to unvaccinated individuals. In Omicron breakthrough cases, a reduction in infectious VL was only observed in boosted but not in fully vaccinated individuals compared to unvaccinated individuals. 

Analyzing real-world data, we have already shown that complete vaccination with the currently available vaccines protects against the transmission of SARS-CoV-2, including the Delta variant [11,12]. Using data from the largest German Public Health Department, Cologne (North Rhine-Westphalia), we now investigated breakthrough infections in booster-vaccinated infected persons (IPs) to close contacts when compared to an unvaccinated control group (UCG).

## 2. Materials and Methods

### 2.1. Study Design and Population

**Figure 1:**
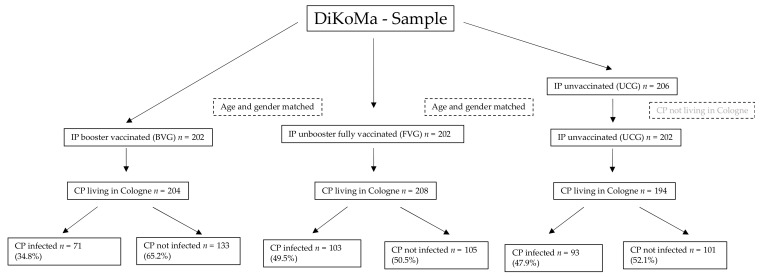
Flow chart of the study population. BVG = booster-vaccinated group; FVG = fully vaccinated group; UCG = unvaccinated control group; IP = infected person; CP = contact person.

Data were collected from 17 December 2021 to 6 January 2022—a period during which the Omicron variant is assumed to have been the most prevalent strain (between approximately 20% in calendar week 50/2021 up to approximately 80% in calendar week 1/2022 [13]). In this period, we identified 206 unvaccinated infected persons ≥ 18 years of age within the DiKoMa registry (Figure 1; [14]). Four of them were excluded who only had contacts outside of Cologne, our area of responsibility. Each patient in the UCG was randomly matched 1:1 by age and sex with a booster-vaccinated SARS-CoV2-positive patient (booster-vaccinated group [BVG]; *n* = 202) and fully vaccinated, not boosted SARS-CoV2-positive patient (unboosted, fully vaccinated group [FVG]; *n* = 202). 

Booster vaccination was assumed [15] if a second mRNA vaccine dose was administered after a first Ad26.COV2.S dose (*n* = 25; 12.38%) or if a mRNA vaccine dose was administered either after two doses of mRNA dose (*n* = 140; 69.31%) or Vaxzevria (*n* = 8; 3.96%) or Vaxzevria combined with a mRNA vaccine (*n* = 29; 14.36%). Only persons who received the booster vaccination at least 2 weeks prior were integrated. On average, booster vaccination was given 32.5 ± 21.8 days prior (between 14 to 154 days). 

Fully vaccinated, not boosted was assumed [15] if the last dose was received more than 3 months ago (two doses of the viral vector vaccine Vaxzevria *n* = 4, 1.98%; one dose of Vaxzevria plus one dose of mRNA vaccine *n* = 27, 13.36%; two doses of mRNA vaccine *n* = 171; 84.65%). 

### 2.2. Data Analyses

The total number of each contact per IP, the total number of infected contacts per IP and the infected contacts relative to the total number of contacts per IP were analyzed. Means and standard deviations were described. As there was no normal distribution, Kruskal–Wallis non-parametric ANOVA and median test were used to determine the significance levels between the three groups. The significance level was set at *p* ≤ 0.05 (SPSS 28.0; IBM, Armonk, NY, USA).

## 3. Results

**Figure 2:**
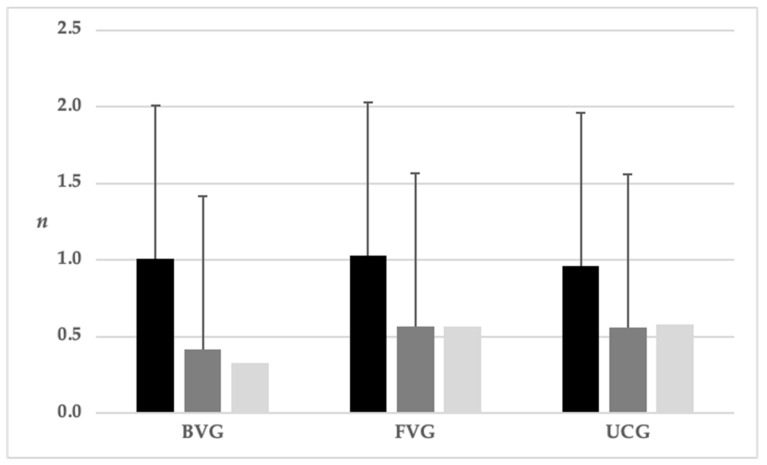
Means and standard deviation of total contacts (black) and infected contacts related to the total number per infected persons (dark grey; median light grey) in the booster-vaccinated group (BVG) vs. fully vaccinated group (FVG) vs. unvaccinated control group (UCG; *p*-values see Table 1).

In both groups, 54.5% were female. Age, number of total and infected contacts and the number of infected contacts relative to the total number of contacts per IP are shown in Table 1. Additionally, the number of total and the number of infected contacts relative to the total number of contacts per IP in all three groups are shown in Figure 2. 

Overall, the UCG had the highest number of infected contacts to the total number of contacts. The comparison of mean values only showed a trend with regard to possible group differences (*p* = 0.054). Taking the medians into account, booster-vaccinated persons had the lowest rate of infected contacts to total contacts compared to the fully vaccinated and unvaccinated groups. There was no difference between the fully vaccinated and the unvaccinated group (Table 2).

## 4. Discussion

Based on these real-life data, the transmission of the Omicron variant has a 25.0% (mean) and 42% (median) lower occurrence in booster-vaccinated patients with breakthrough infection as in an unvaccinated control group. No difference in terms of transmission occur between fully vaccinated and unvaccinated infected persons. Thus, these real-world data confirm the observations of previous studies.

However, there are some limitations to this analysis. The main point besides the small number of included cases is the fact that an infection with the Omicron variant was made as a conclusion by analogy. Due to the increasing number of cases, sequencing was only carried out selectively. Following the guidelines of the German Corona-Surveillance Ordinance, 5–10% of all incoming samples are sequenced [16]. The largest laboratory in Cologne announced and still announces the results of their sequencing via Twitter. In total, 34 of our total cases fell into the phase where Omicron accounted for about 20% of the viral variants, 182 cases accounted for about a third and the remaining 390 cases fell into the phase where 75 to 80% Omicron was detected [17]. Similarly, in the compilation of the Robert Koch Institute for the calendar week 51/2021 (start 20.12.), based on selected sequencing for North Rhine-Westphalia, the occurrence of Omicron was 20.9%, in calendar week 52/2021 it was 52.4% and in calendar week 1/2022 it was 75.8% [13]. Even though we did not have any sequencing data available, it can be assumed that the majority had this variant. Certainly, from today’s point of view, the detection of possible subvariants should also take place; however, at that time, the BA.1 subvariant from Omicron was leading in Germany. 

The number of contacts indicated is also correspondingly low. Of course, we can only speculate, but from 16 December 2021 in North Rhine-Westphalia, closures of public events, e.g., New Year’s Eve parties, were again made depending on the incidence figures. Especially for the unvaccinated, visits to restaurants and participation in sporting, cultural leisure activities were prohibited and contacts in private were limited to a maximum of two households. It can therefore not be ruled out that in some cases, contact persons were not even indicated. It is possible that people did not even get tested after contact with or without symptoms in order to avoid quarantine. However, we cannot exclude the possibility that other reasons also led to more or less transmission. It can be assumed, for example, that adherence to rules, but also the indication of possible contact persons, is higher in vaccinated persons than in unvaccinated persons. Additionally, the vaccination status of the contact persons was not considered. Due to the design, no sociodemographic factors and/or other possible influencing factors, e.g., symptoms, could be taken into account.

## 5. Conclusions

Despite the mentioned limitations, our real-world data confirm the laboratory findings of Puhach et al. [6]: only a booster vaccination may reduce transmission in the immune escape variant Omicron. To what extent this is an expression of an actual booster effect or ultimately a refreshment of the immunity that has been reduced over time remains open. In addition, the high dynamics and mutation tendency of SARS-CoV2 requires rapid adaptations of the vaccines and/or broader therapeutic schemes [18,19].

## Figures and Tables

**Table 1:** Total contacts, total infected contacts and related to total number per infected persons (IP) in the booster-vaccinated group (BVG) vs. the fully vaccinated group (FVG) vs. the unvaccinated control group (UCG).

	Group (*n*)	Mean	SD	*p*-Value *	Range	Median	*p*-Value **
Age (yrs.)	BVG (202)	36.80	12.90	1.000	18–93	34.0	1.000
	FVG (202)	36.70	12.60		18–75	34.0	
	UCG (202)	36.90	13.00		18–96	34.0	
Number of contacts per IP ^†^	BVG (202)	1.01	1.79	0.680	0–11	0	0.456
	FVG (202)	1.03	1.75		0–15	0	
	UCG (202)	0.96	1.80		0–18	0	
Number of infected contacts per IP ^‡^	BVG (83)	0.86	0.96	0.115	0–5	1	0.041
	FVG (95)	1.08	1.07		0–5	1	
	UCG (86)	1.08	0.91		0–3	1	
Number of infected contacts to total number of contacts per IP ^‡^	BVG (83)	0.42	0.42	0.054	0–1	0.33	0.004
	FVG (95)	0.57	0.44		0–1	0.57	
	UCG (86)	0.56	0.43		0–1	0.58	

* Calculated with Kruskal–Wallis non-parametric ANOVA. ** Calculated with the median test. ^†^ Persons who did not indicate close contacts were also integrated in order not to distort the number; ^‡^ only taken into calculation if close contacts were indicated; yrs. = years, IP = infected person.

**Table 2:** Pairwise comparison of total infected contacts related to the total number per infected persons between BVG (booster-vaccinated group) vs. FVG (fully vaccinated group) vs. UCG (unvaccinated control group).

Sample	*p*-Value *	*p*-Value after Bonferroni Correction *
BVG vs. UCG	0.008	0.025
BVG vs. FVG	0.002	0.005
UCG vs. FVG	data	data

* Calculated with the median test.

## Data Availability

The data used and analyzed in the current study involve sensitive patient information and indirect identifiers.
